# A perspective on the PDB’s impact on the field of glycobiology

**DOI:** 10.1016/j.jbc.2021.100556

**Published:** 2021-03-18

**Authors:** James H. Prestegard

**Affiliations:** Complex Carbohydrate Research Center, University of Georgia, Athens, Georgia, USA

**Keywords:** glycoproteins, glycans, crystallography, cryo-EM, NMR, computational modeling, CAZy, Carbohydrate-Active enZYmes, EPO, erythropoietin, ER, endoplasmic reticulum, Gal, galactose, Glc, glucose, HS, heparan sulfate, IgG, immunoglobulin G, LAR, leukocyte common antigen-related protein, Man, mannose, Neu5Ac, *N*-acetylneuraminic acid, OGA, O-GlcNAc hydrolase, OGT, O-GlcNAc transferase, OST, oligosaccharide transferase, PDB, Protein Data Bank, SNFG, Symbol Nomenclature for Glycans

## Abstract

Structures deposited in the Protein Data Bank (PDB) facilitate our understanding of many biological processes including those that fall under the general category of glycobiology. However, structure-based studies of how glycans affect protein structure, how they are synthesized, and how they regulate other biological processes remain challenging. Despite the abundant presence of glycans on proteins and the dense layers of glycans that surround most of our cells, structures containing glycans are underrepresented in the PDB. There are sound reasons for this, including difficulties in producing proteins with well-defined glycosylation and the tendency of mobile and heterogeneous glycans to inhibit crystallization. Nevertheless, the structures we do find in the PDB, even some of the earliest deposited structures, have had an impact on our understanding of function. I highlight a few examples in this review and point to some promises for the future. Promises include new structures from methodologies, such as cryo-EM, that are less affected by the presence of glycans and experiment-aided computational methods that build on existing structures to provide insight into the many ways glycans affect biological function.

## Glycobiology

Glycobiology is the study of how glycans, also called carbohydrates or oligosaccharides, result from, or have an impact on, a wide array of biological processes (1). Their impact is broad, with glycans occurring on the surfaces of most cells in addition to being excreted into the environment and incorporated into structural materials that constitute most of the biomass on earth (2). Many glycans are covalently attached to proteins, making them glycoproteins. It is estimated that, among all proteins in the Swiss-Prot database, between 20% and 50%, are glycosylated (3, 4); among eukaryotes (about one-third of the proteins in the database), the percentage is likely to be much higher (5, 6). In addition to glycoproteins, numerous other proteins bind glycans in the process of transforming them for a source of energy or for substrates needed to build other biological macromolecules, including the structural carbohydrates of plants and microbes. Some proteins also bind to glycans on cell surfaces to initiate various signaling and adhesion events, and pathogens, both bacterial and viral, have proteins that bind to cell surface glycans as a step in the infection process. This adds to the biomedical importance of glycans and has raised the interest of the pharmaceutical industry in both glycoproteins and glycomimetic glycans (7).

Glycans are structurally very diverse, more so than polypeptides and nucleic acids of comparable size. Although the number of residue types (sugars) making up glycans is similar to the number of amino acid types making up proteins, residues can be linked in multiple ways and they can exist as branched and linear oligomers. Differences between some glycan residues seem subtle. For example, glucose (Glc), mannose (Man), and galactose (Gal) are all composed of six-membered pyranose rings with exactly the same composition, C_6_H_12_O_6_, from which the name carbohydrate is derived. However, structural consequences associated with differences in chirality are significant. Also, unlike proteins and nucleic acids, glycan synthesis is not template driven but occurs *via* the combined action of hundreds of enzymes that add or remove individual residues. As a result, glycoproteins seldom carry a single type of glycan, making them heterogeneous even at the single site level. These properties have certainly impeded characterization of glycans and perhaps the interest of the broader scientific community in studying of how glycans influence biological processes.

Recently, some steps have been taken to make glycobiology more accessible to a broad audience and to attract a new generation of scientists who will tackle challenging glycobiology problems. One step is a text, now in its third edition, *Essentials of Glycobiology*, that begins with a useful historical review of the field (1). The authors and editors receive no financial benefit from sales of this book, and they have arranged to have electronic versions freely available through the NCBI Bookshelf (https://www.ncbi.nlm.nih.gov/books/NBK310274/). Another step is the adoption of a set of symbols for residues that make up glycans. Chemical structures are, of course, important to understanding glycan interactions with proteins and other glycoconjugates, but in many cases replacing atomic depictions of sugar residues with simple symbols is sufficient to show differences in glycan structures and place glycans in the larger biological contest where they function. Symbol Nomenclature for Glycans (SNFG) provides different symbols for each type of sugar (8). Software facilitating the depiction of these symbols in three dimensions has also been devised (9).

**Figure 1:**
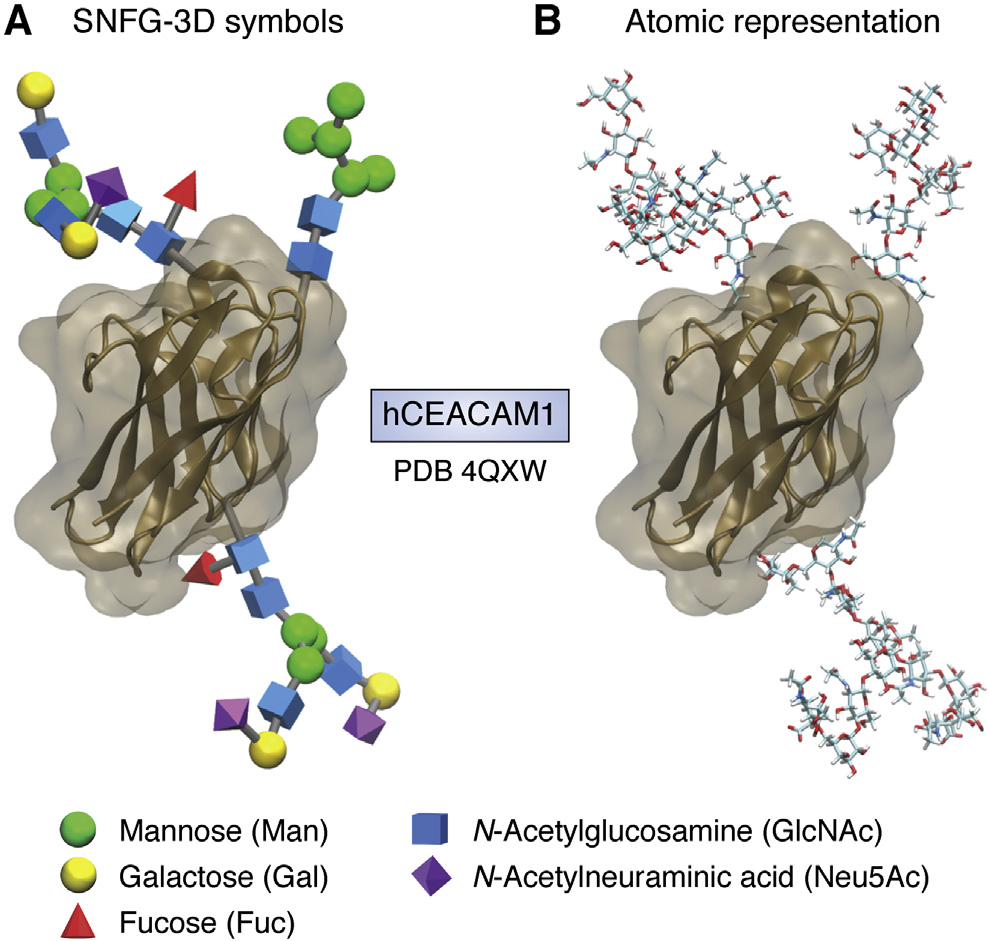
**N-terminal domain of hCEACAM1 (4QXW).** N-glycans are shown in SNFG-3D symbols (*A*) and full atomic representations (*B*). Spatial occupancies of protein and glycans are equally well represented in both depictions. SNFG, Symbol Nomenclature for Glycans.

Figure 1*A* illustrates the use of the 3D symbols (http://glycam.org/3d-snfg) to depict a possible spatial arrangement of glycans in a particularly heavily glycosylated protein, the 11-kDa *N*-terminal domain of human carcinoembryonic antigen–related cell adhesion molecule 1. For comparison, the same structure is depicted in Figure 1*B*, showing the atomic detail of glycans. The domain has three *N*-glycosylation sites in which glycans are linked to the side-chain nitrogen of an asparagine in a consensus amino acid sequence, NXS/T. Three of the glycans frequently found on this domain are depicted, one high Man glycan composed of two GlcNAc residues (blue squares) and five Man residues (green circles) and two complex glycans that have additional Gal residues (yellow circles), fucose residues (red triangles), and a particular type of sialic acid residue, *N*-acetylneuraminic acid (Neu5Ac, purple diamonds). These glycans have been modeled onto the crystal structure of a nonglycosylated version of the protein (Protein Data Bank [PDB] ID: 4QXW) using computational modeling (10). As with other figures in this article, this figure has been made with readily available visualization tools (11, 12). The spatial extension of the glycans is well represented in both depictions, but residue types are more easily recognized using SNFG symbols. It is striking how much space glycans can occupy compared with the underlying protein in this example. In many cases, SNFG symbols now appear in the 3D views supplied when first accessing a glycoprotein deposition in the PDB.

## Structural glycobiology and the PDB

Most of the structural biology community may well view glycobiology as a recent entry into the world of structure-based investigation. Nothing could be further from the truth. Emil Fischer, a 1902 recipient of the Nobel prize in Chemistry, developed the Fisher projection as means of describing the chirality of successive carbons in the simple sugars that make up glycans. He also introduced the lock and key model of enzyme action while contemplating how enzymes ferment glucosides, in which the simple sugar, Glc, is linked to other sugars or aglycons (13). Interactions with the PDB also started surprisingly early; 10 of the first 51 structures deposited in the PDB (starting in November of 1977) came from fiber diffraction studies of polysaccharides. Since that time, with appropriate emphasis on macromolecular structure, proteins and nucleic acids have dominated depositions. However, glycan structures persist as ligands in the binding sites of proteins and as the covalent adducts that turn proteins into glycoproteins. The percentage of protein depositions in the PDB annotated with the structure description “glycoprotein” has risen steadily from 0.2% in the 1990 to 2000 period to 0.5% in the 2000 to 2010 period and to 0.9% in the 2010 to 2020 period. The percentage annotated with “saccharide” as a chemical component type (mostly bound ligands) has remained fairly constant between 1.2 and 1.5%.

Some of the difference between deposition statistics and potential impact may reside in the difficulty in retrospectively identifying structures that contain glycans. For years, naming of glycan residues and the use of keywords to identify the glycan content were left entirely up to authors of the depositions. Much of this has now been corrected through the use of improved deposition and remediation tools for carbohydrates (https://www.wwPDB.org/documentation/carbohydrate-remediation). Also, there are now software search tools that use either atom connectivity data (14) or actual structural footprints https://dev.glycam.org/portal/gf_home/ (15) to find relevant structures in the PDB. A recent application of one of these tools suggests that the percentage of depositions containing a carbohydrate moiety is actually 7.7% (14).

There are some real difficulties in producing structures of glycoproteins. For X-ray crystallography, producing suitable crystals can be problematic. Native glycosylation is very heterogeneous; attached glycans are very mobile, and they can actually dominate a glycoprotein structure as seen in Figure 1. The heterogeneity, mobility, and bulk of glycans can all contribute to a failure to crystallize (16). Fortunately, methods for engineering glycans have advanced, and producing glycoproteins with less extended and more homogeneous glycosylation now exist (17, 18). For NMR, most structural studies depend on uniform isotope labeling with ^15^N and ^13^C. This is economical for proteins that can be expressed in bacterial cultures which synthesize all amino acids from simple substrates (*e.g.*, ^15^NH_4_Cl and ^13^C-Glc), but for glycoproteins expressed in mammalian cells, which produce near-native glycosylation, this can be very expensive, and perdeuteration needed to work on larger proteins is usually not possible. Fortunately, there are advances that make expression in mammalian cells less costly (19), and there are resonance assignment strategies that do not depend on uniform isotope labeling (20). NMR also excels when bound ligands are of interest. Glycan ligands tend to have relatively low affinities, something that is often compensated by multivalency in biological contexts. For crystallography, this leaves sites in crystals unoccupied, but weak binding ligands are not a problem for NMR-based investigations.

Importantly, cryo-EM structures, which have come on the scene more recently, do not require crystallization and provide a possible route to increased numbers of structures of glycoproteins, particularly large ones (21). For the last 6 months, nearly 15% of the deposited electron microscopy structures have a structure description of glycoprotein. With this new technology, and despite the challenges to producing structures with X-ray and NMR methods, there are now many structures deposited in the PDB that have had an impact on our understanding of how glycans influence biological function. In this review, I will touch on a few of these examples.

## Erythropoietin—a glycosylated recombinant pharmaceutical

Erythropoietin (EPO) is a heavily glycosylated human protein hormone responsible for inducing differentiation of bone marrow erythroid progenitor cells to form red blood cells. The native protein has three *N*-glycosylation sites, easily identified by their conserved consensus sequence (NXS/T), and one O-glycosylation site. These are all at least partially occupied with a heterogeneous array of glycans. EPO was the first biopharmaceutical produced as a recombinant protein in mammalian cells, being approved by the FDA for the treatment of anemia in 1989. It was also the first glycosylated recombinant protein to surpass annual sales of $1 billion (22). The importance of glycosylation was determined early in its development (23), and efforts immediately began to alter glycosylation to improve activity and extend lifetime in the blood stream. The presence of complex glycans, particularly those terminated with sialic acid, was found to do both, extending lifetime and activity by almost an order of magnitude.

Structure certainly played a role in this development. There are only two structures of EPO in the PDB, both deposited relatively early in the history of the PDB. One was determined by NMR methods (1BUY, deposited in 1998) (24) and one by X-ray crystallography, in which EPO is complexed with its dimeric receptor (1EER, also deposited in 1998) (25). Interestingly, neither of these contains coordinates for glycans. All three of the potentially glycosylated asparagines in EPO were replaced with lysine to produce a soluble analog that could be expressed in a nonglycosylating *Escherichia coli* culture. Although the receptor was expressed in a potentially glycosylating yeast culture, the asparagine in its single *N*-glycosylation site was mutated to glutamine to minimize interference with crystal formation. The structures provided insight into how binding may stimulate receptor signaling. Both receptor and EPO appear to alter structure on complex formation.

**Figure 2:**
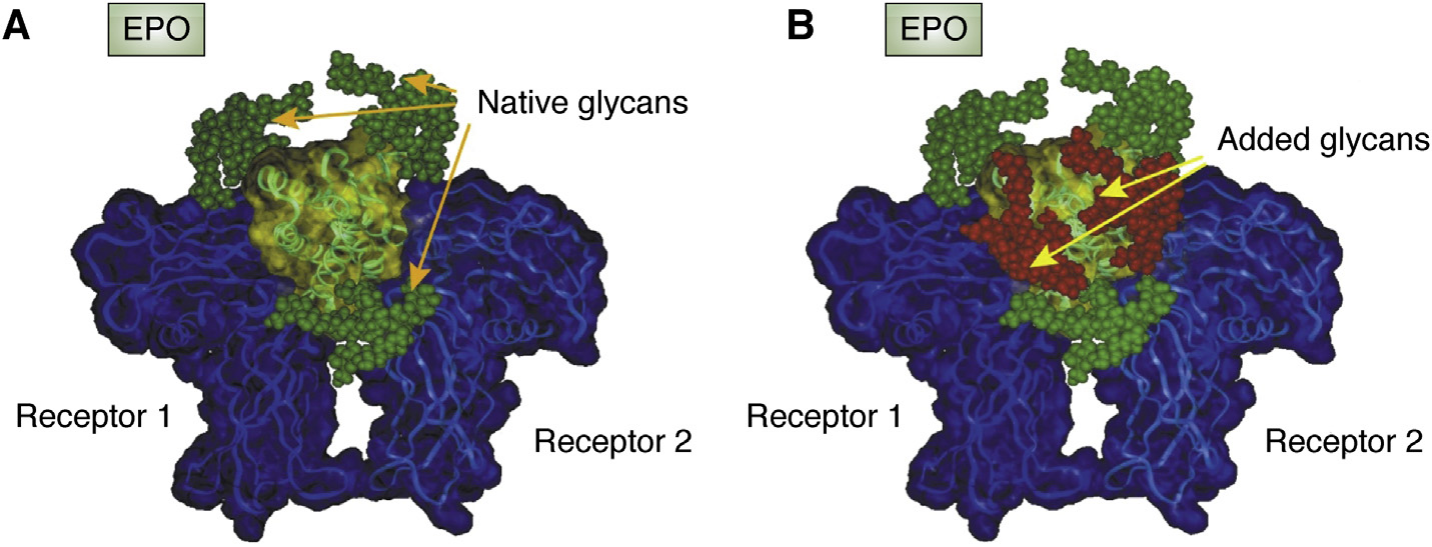
**Comparison of EPO with native glycans and added glycans.** The crystal structure is of the nonglycosylated extracellular domain of the EPO receptor bound to an EPO analog (1EER). A structure of a tetra-antennary glycan terminated with sialic acids was produced by molecular modeling and attached to the original sites (*A*) or new N-linked glycosylation sites (*B*) on the EPO structure. Structures are EPO (*yellow*/*green*), EPO receptors (*blue*), original three N-linked glycans (*green*), and new glycans (*red*). Reproduced with permission, Nature Biotechnology, 21:417, 2003. EPO, erythropoietin.

Despite the lack of glycans in these structures, they provided a basis for understanding the potential role of the glycans and how additional glycans might improve efficacy of EPO. Figure 2 shows how Elliott *et al.* (23) modeled in native glycans to identify a region in the protein sequence where an additional *N*-glycosylation sequon could be added without inhibiting the receptor interaction but potentially increasing the protein’s stability and resistance to clearance from the blood stream. Since that time, these same structures have been used repeatedly to facilitate the design of new glycosylated species and rationalize their effects on efficacy of the drug. Much effort has gone into producing constructs with homogeneous glycosylation using purely chemical methods (26, 27) and enzyme facilitated methods (28). Glycans clearly stabilize the structure, improve solubility, and particularly with glycans terminated in sialic acid, prolong residence in the blood stream. The latter presumably occurs because of reduced clearance through the asialoglycoprotein receptor. A certain level of intrinsic disorder in the binding elements of EPO also appears to be important to its ability to interact with other receptors and elicit other physiological responses (22). Glycosylation may well play a role in regulating structures of these regions as well.

Modeling glycans into proteins in which glycosylation sites have been removed by mutation, or into proteins where there is simply insufficient electron density to place glycan residues experimentally, continues today. Recent examples of addition of glycans by modeling include the spike proteins of the coronavirus SARS-CoV-2, where heavy glycosylation obscures sites that might have been used for antibody development (29, 30).

## Glycans and antibody function

**Figure 3:**
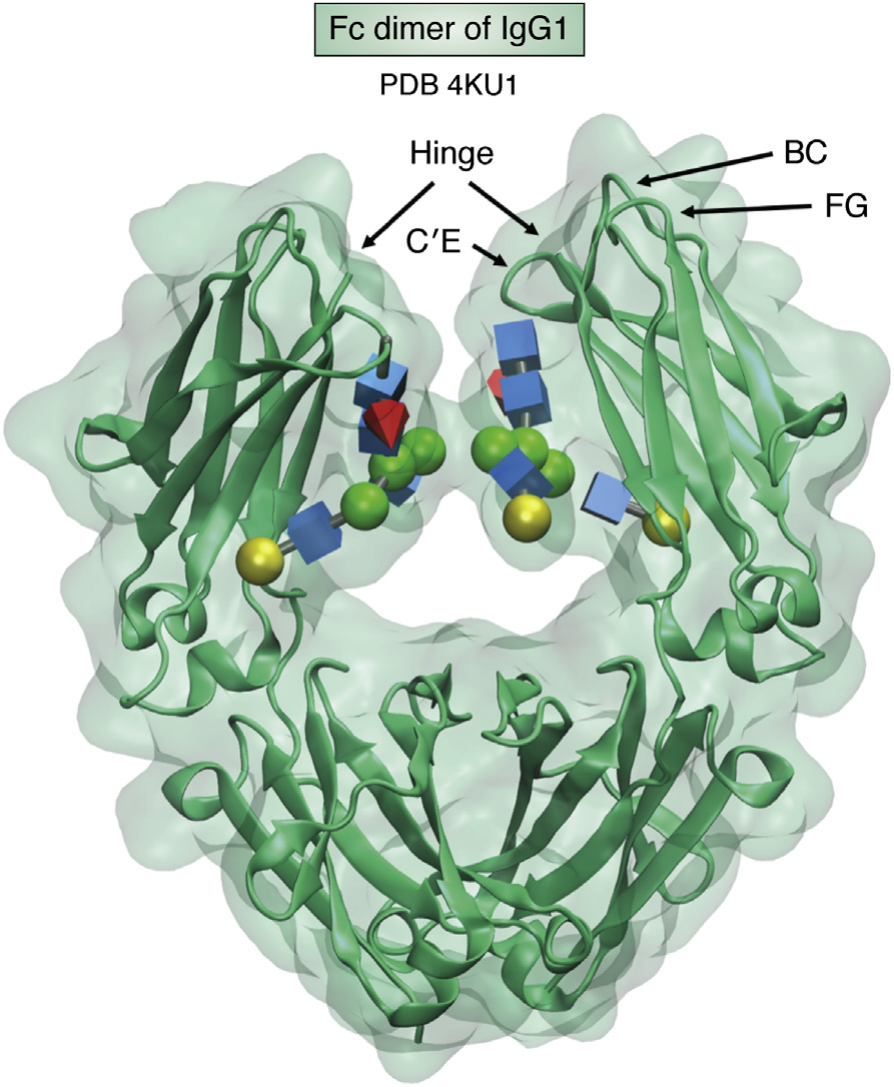
**Fc dimer of IgG1 depicted using PDB deposition 4KU1.** Glycans are shown in 3D-SNFG symbols. The Gal terminating the 6 branch of one monomer is shown as a *yellow sphere* at the center left, and the N-acetylglucosamine (GlcNAc) terminating the 3 branch of the other monomer is shown as a *blue cube* near the center of the cavity. PDB, Protein Data Bank; SNFG, Symbol Nomenclature for Glycans.

Although glycans on the surface of glycoproteins, being flexible and heterogenous, often work against crystal formation, there are exceptions. The dimer formed by the fragment crystallizable (*F*c) region of human immunoglobulin G (IgG) antibodies is one. The *F*c region found at the C-terminus of the heavy chain of IgG antibodies has a single *N*-linked glycan, which proves to be reasonably homogeneous when isolated from pooled human serum. The dominant glycans are core-fucosylated biantennary structures with zero, one, or two Gal residues terminating the branches (31). In 1976, Huber *et al.* (32) reported a crystal structure of *F*c; it was refined and deposited in the PDB in 1981 (1FC1) (33). This is possibly the first glycoprotein deposited in the PDB. Many structures of this fragment have been deposited since. Enzymatically engineering glycans has improved resolution substantially, but the general placement of glycans has remained the same. Depicted in Figure 3 is a more recent structure (4KU1 (34)). The glycans are inside a cavity formed between the two *F*c chains of the dimer and are closely packed against the protein surface. Immobilization of the glycans in this way clearly contributes to the quality of the structure.

The glycans are linked to asparagine 297 near the top of the structure as depicted in SNFG-3D symbols. There is a mobile disulfide linkage forming a hinge between monomers near the top (not resolved in this crystal structure). The rest of the heavy chains extend out from this region to interact with another pair of chains to form the Fab domains responsible for antigen binding. The hinge region, along with BC, C’E, and FG loops, is also the area involved in binding the Fc gamma receptors that turn antigen binding into a physiological response.

Given the proximity of glycans to the hinge region, it is not surprising that early work found that producing IgG antibodies without glycosylation resulted in loss of an ability to activate complement and induce cellular toxicity (35) but did not affect antigen binding or binding of protein A (a bacterial protein that recognizes structural elements of *F*c near the outer midpoint of the depicted structure). In subsequent work, it became clear that even small changes in glycan structure had substantial physiological effects. Absence of terminal Gal residues is associated with inflammation, and the addition of a sialic acid to the terminal Gal residues produces an anti-inflammatory molecule that appears to be the active component in intravenous IgG treatments for severe cases of rheumatoid arthritis (36). The exclusive use of a particular sialic acid to terminate IgG Gal residues, Neu5Ac, is characteristically human. Other mammals, including other primates, use both Neu5Ac and *N*-glycolylneuraminic acid. Specific recognition of Neu5Ac is important for glycoproteins implicated in diseases outside those associated with immune response (37), and an understanding of the role of sialic acids in immune response may contribute to understanding those diseases as well.

The mechanism by which changes in physiological function of IgGs occurs is still an important target of investigation (38). Chimeric constructs having an *F*c component, as well as the antibodies themselves, now dominate sales of the pharmaceutical industry, and having a rational approach to altering glycan composition could have an enormous impact. Structures of *F*c having engineered complements of glycans, or in complex with various Fc gamma receptors, are providing snapshots of interactions and structural changes in the hinge region that corelate with glycan substitutions. There are now more than 100 such depositions in the PDB. There is clearly considerable variation in the conformation of the receptor binding region. However, the picture may be more complex than static snapshots can provide. Solution-based methods such as NMR and SAXS suggest that the region may dynamically sample these conformations (39). Also, the glycans themselves do not stay in the position depicted. Instead, they transiently sample extended conformers where enzymatic modification of glycans can occur (40). Ultimately, shifts in distributions in response to changes in glycan composition may be a better description of how glycans affect IgG function.

## Glycans as ligands in protein structures

Far more abundant among structures deposited in the PDB, than glycans covalently attached to glycoproteins, are glycans as ligands of proteins that bind glycans, modify glycans, or add glycans to other proteins. The first example of an enzyme with a bound substrate was hen egg white lysozyme, deposited in 1979 as one of the first 65 structures in the PDB. Lysozyme is a hydrolytic enzyme with homologues produced by many animals as an antibacterial agent. The 1979 structure (9LYZ) was a complex with a trisaccharide, containing *N*-acetylmuramic acid (MurNAc) and GlcNAc, MurNAcβ1-4GlcNAcβ1-4MurNAc. Mechanistically, there had been much speculation about capturing a transition state with a distorted MurNAc pyranose ring. The crystal structure failed to show this distortion (41). Many structures of lysozymes followed (now nearly 1000 structures from various species). However, direct experimental evidence for a distorted transition state remains elusive (42). Resolution of detailed mechanistic questions may fall to computational work that builds on these many high-quality crystal structures (43).

In general, enzymes that build or degrade glycans represent a major class of protein structures deposited in the PDB. There is, in fact, a separate database, the Carbohydrate-Active enZYmes (CAZy) database (http://www.cazy.org), that assembles domains of these proteins into families. This was originally based on sequence and evolutionary relationships; however, it has evolved to incorporate biochemical data and structural data from the PDB. Early on, it provided a resource for finding enzymes, systematically naming enzymes, and displaying bound glycan ligands. It is now apparent that families are as much structurally related as they are sequence related (44). There are now nearly a million modules classified into families (a single modular protein can be classified into many families), and there are nearly 10,000 PDB IDs associated with these modules. While the CAZy database does classify noncatalytic, glycan-binding modules, there are also databases devoted specifically to proteins containing only these modules, namely lectins. The UniLectin3D database (https://www.unilectin.eu/unilectin3D) classifies more than 2000 PDB structures into 35 families that share protein folds (45). These databases have become important tools for understanding how structure relates to function. Both are highly dependent on the PDB, with CAZy linking its entries to the PDB and UniLectin3D actually classifying entries based on structural folds.

It seems appropriate to highlight one recent example of structures providing insight into the function of a carbohydrate active enzyme (actually a pair of enzymes). The pair is the *O*-GlcNAc transferase (OGT) and the *O*-GlcNAc hydrolase (OGA) responsible for respectively adding and removing a single GlcNAc residue at serine and threonine sites that are often phosphorylated in higher organisms. This unusual addition of a single sugar with no further extension was not discovered until 1984 because of the size, lack of charge, and lability of the addition (46). However, it proves very ubiquitous and functionally important to a host of processes including nutrient sensing, response to stress, cell division, and transcription (47). Moreover, it dispelled the common assumption that glycosylation was a process relegated to extracellular proteins and proteins involved in, or destined for, the secretory pathway. The modification occurs in both the cytosol and the nucleus. Its competition with phosphorylation has suggested that it is a master regulator of many signaling processes, but in contrast to the hundreds of kinases and phosphatases involved in phosphorylation, there is just a single pair of glycosyltransferase and glycosyl hydrolase enzymes. This raises many questions about how these enzymes find their many targets and to what extent modifications are selective. The structure of these two enzymes provides a starting point for answers to these questions.

**Figure 4:**
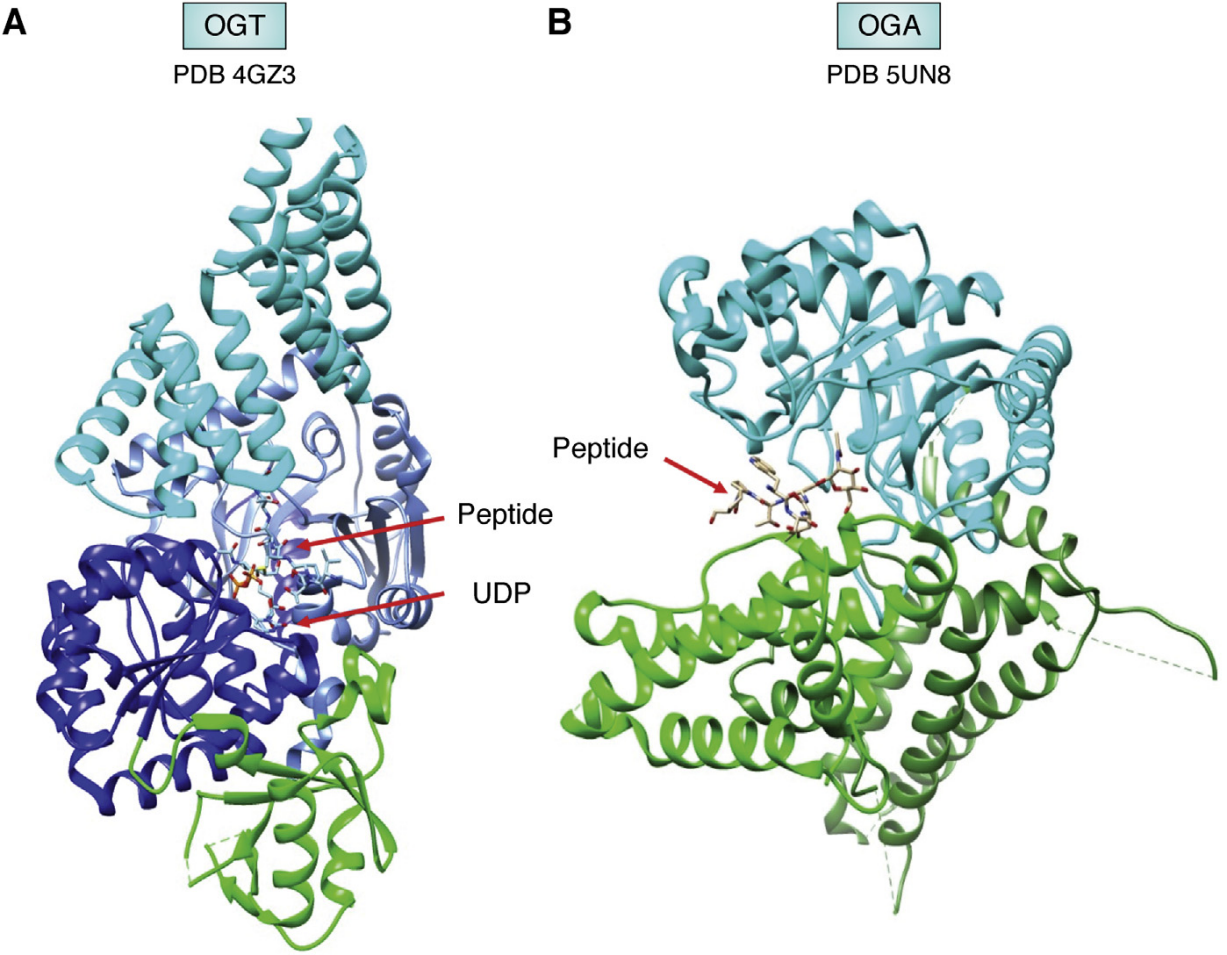
**Structures of O-GlcNAc enzymes.***A*, OGT (4GZ3) catalytic units are *blue* and *cornflower blue* with the donor product (UDP) and acceptor peptide shown in *stick figures*. The positively charged domain inserted between two parts of the catalytic domain is in *green* and the tetratricorepeat domain is in *cyan*. *B*, an OGA structure (5UN8) is shown with the O-GlcNAc modified peptide from p53 inserted into the catalytic domain of one monomer of the dimeric structure (*cyan*). The peptide makes additional contacts with the stalk region of the second monomer (*green*, as opposed to *forest green*, for the first monomer). OGT, *O-*GlcNAc transferase.

The structure of OGT is depicted in Figure 4*A* (48). The catalytic domain (in blue and cornflower blue) may look similar to a number of other glycosyltransferases. It belongs to the family of GT-B glycosyltransferases and consists of a pair of Rossman folds forming a binding site for the sugar donor, UDP–GlcNAc. It is, however, unusual in that it has a second activity, a proteolytic cleavage of HCF1 to produce a mature cell cycle coregulator (49). Also, there is a highly negatively charged domain between the two Rossman folds (green) that may suggest interactions with nucleic acids, and the *N*-terminal domain (cyan) is a series of tetratricopeptide repeats, domains that are well-known for their protein–protein interactions.

Recently, two groups have produced structures of human OGA (50, 51). The one depicted in Figure 4*B* (5UN8) crystallized as a dimer with an O-GlcNAc-containing peptide from p53. The catalytic domain of one monomer is shown in cyan along with its stalk domain in forest green. The stalk domain of the other monomer is shown in green. Interestingly, both the catalytic domain of one monomer and the stalk domain of the other monomer make contact with the substrate. The extended surface contacts suggest a mechanism whereby activities toward certain glycosylated peptides may be enhanced. These depictions of surface contacts are proving useful in the design of a number of OGA inhibitors (52). Although there are structures of both OGT and OGA with peptides from potential substrates bound, a full understanding of target selection may await structures of complexes containing the actual protein substrates or complementary data from techniques that can identify regions involving more transient protein–protein contacts.

## Large protein assemblies from cryo-EM

Although the structural detail offered by high-resolution X-ray structures of individual proteins remains essential for mechanistic studies and inhibitor design, other challenges remain that require new technology. Advances in cryo-EM technology allowing production of large structures with sub 3Ǻ resolution is revolutionizing many areas of structural biology, including structural glycobiology. Without the requirement for crystallization, the mobility and heterogeneity of glycosylation imposes few impediments. In many cases, sufficient electron density is not seen for glycans beyond the first glycan attached, but protein structures can be produced and glycans added computationally. Also, because the size is more of an advantage than disadvantage, many of these structures are of multiprotein complexes, including the now numerous structures of whole virus particles, all of which are heavily glycosylated.

The impact of cryo-EM on glycoscience is, however, best illustrated with a nonviral example of multiprotein assemblies, namely the oligosaccharide transferases (OSTs) which are embedded in the endoplasmic reticulum (ER). The *N*-glycans, that play important roles in protein folding and extracellular recognition events, are not added stepwise starting with the first GlcNAc attached to the asparagine side chain of the NXS/T sequons. Instead, they are first transferred by the action of OST enzymes as a large oligosaccharide (Glc_3_Man_9_GlcNAc_2_) from a dolichol pyrophosphate donor anchored to the luminal side of the ER. From there, they are trimmed and modified before transfer to the cisternae of the Golgi apparatus for further modification and eventual secretion. The study of complexes embedded in membranes, whether membrane isolates (for EM tomography) or membrane mimetics (for single-particle EM), is an area where cryo-EM approaches excel (53, 54). One of the earliest medium-resolution cryo-EM articles on an OST focused on interactions with the ribosome. It documented the interaction of an OST with the translocon, another multiproton complex, that includes Sec61 which provides a tunnel through the ER (55).

*N*-glycan sequons are not glycosylated to an equal extent, and the extent clearly varies with the context in which the sequons are found. Part of the challenge in understanding the origin of variations in glycosyl addition arises from mammals having two OSTs, one that operates cotranslationally (OST-A) and one that operates post-translationally (OST-B). Both OSTs are multiprotein complexes, having catalytic units (STT3A and STT3B) that differ slightly in sequence. These units are complexed with 5 other proteins that they share, but OST-A has an additional protein that appears essential for interaction with the ribosome, DC2, and OST-B has one of two oxidoreductases that facilitate post-translation glycosylation, MAGT1 or TUSC3. The entire assembly of each is embedded in the ER membrane with a cluster of transmembrane alpha helices. Recently, high-resolution structures (3.5 Ǻ) of both OST-A and OST-B were obtained. They provide interesting details explaining why more specific associations with the translocon occur for OST-A than for OST-B (56).

**Figure 5:**
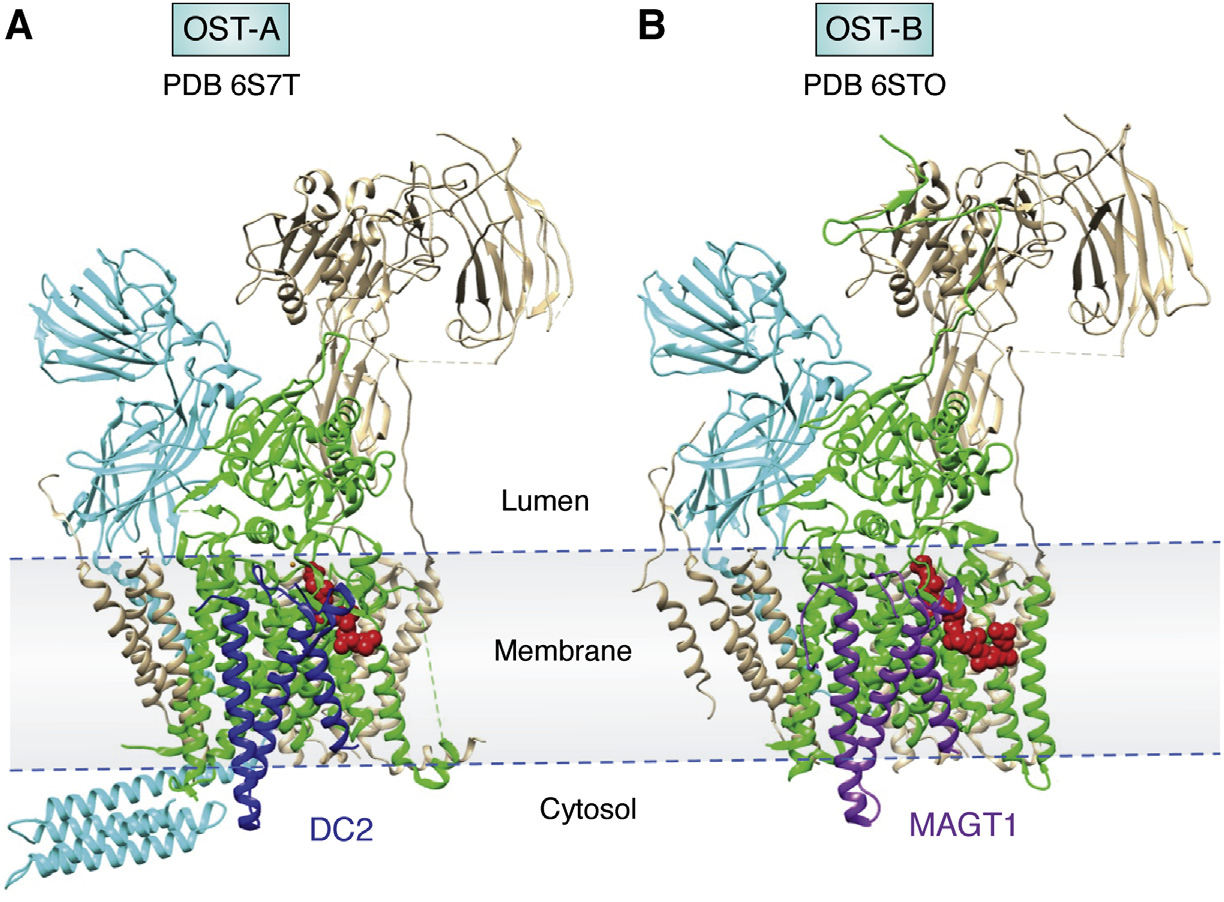
**Cryo-EM structures for oligosaccharyltransferase enzymes.***A*, OST-A (6S7T) is the cotranslational enzyme containing DC2 (*blue*). *B*, OST-B (6S7O) is the post-translational enzyme containing MAGT1 (*purple*). Both enzymes have catalytic units (STT3) shown in *green* and ribophorin-I shown in *cyan*. Both have a dolichol phosphate reaction product shown in *red spheres*. OST, oligosaccharide transferase.

The two human OST assemblies, OST-A and OST-B, are depicted in Figure 5, *A* and *B*, respectively. The respective catalytic units are depicted in green. In both cases, a dolichol phosphate molecule is present (red spheres) identifying the active site. One apparent difference is that the OST-A structure shows a four-helix bundle depicted in cyan at the lower left that extends into the cytosol. This is the C-terminus of ribophorin-I, a protein that is known to interact with ribosomes. This segment is present but disordered and unobservable in OST-B, presumably because of different interactions with STT3A and STT3B. Colored blue in OST-A and purple in OST-B are the transmembrane helices of DC2 and MAGT1 proteins. Despite substantial sequence variations, these helices sit in homologous sites in the OSTs. In OST-A, DC2 would facilitate interaction with the ribosome and the cotranslational addition of an *N*-glycan. In OST-B, the segment of MAGT1 shown has a substantially different sequence and likely inhibits interaction with the ribosome. Instead, the MAGT1 protein has the capacity to disrupt and reform disulfides, something that may be needed in post-translational *N*-glycan additions. Hence, there are clear structural differences that explain why the two OSTs function cotranslationally *versus* post-translationally.

However, there is also much work to be done. There are, for example, several domains in the OST-B and OST-A structures that lack sufficient electron density for structural determination. One is the large *N*-terminal catalytic domain of MAGT1. This would sit on the luminal side of the ER near the dolichol phosphate. There is a crystal structure of a closely homologous domain from the TUSC3 protein (4M91, 72% identity) (57). Positioning it in the structure may clarify how post-translational glycan addition proceeds and whether extensive unfolding is required. Positioning this may require cooperation of scientists using a host of other structural technologies.

## PDB structures leveraged by active site modeling

Despite the tremendous advances in structure determination methodology, there are aspects of glycosylated and glycan processing systems that cannot be captured in experimental structures. The dynamic motions of these systems, the transient binding of ligands and the complete structures of systems that cannot be produced in the amounts or levels of purity required for experimental determination, are a few examples. In these cases, PDB structures provide starting points for building additional structures by homology modeling, for displaying motion in molecular dynamics simulations, and for ligand docking using purely computational methods or computational methods reinforced by limited amounts of experimental data.

One common source of complementary data is NMR. Of course, NMR can provide complete *de novo* structures. In fact, more than 13,000 structures produced by NMR methods have been added to the PDB, many as the result of the Protein Structure Initiative (58). Some of the NMR structures are actually glycoproteins that contain glycans and require these glycans for stability; the first deposited glycoprotein structure by NMR was one of the human CD2 adhesion protein in 1993 (59). More recently, solid-state NMR has come on the scene, providing access to insoluble aggregates of proteins and other amorphous material, which often contain glycans (60). However, because glycoproteins usually require expression in organisms other than *E. coli*, the uniform labeling with ^13^C,^15^N, and ^2^H commonly used for complete structure determination by NMR is often considered impractical, and applications to large glycosylated systems have been slow in coming.

Applications of NMR to locate binding sites for glycan ligands on proteins are more numerous. These applications use very basic experiments where the positions of cross-peaks in two dimensional plots are perturbed by ligand addition (chemical shift perturbation), providing binding-site locations. Resonances from ligands can also suffer intensity changes on irradiation of protein protons with Rf identifying protein-binding epitopes (saturation transfer difference experiments), and cross-peaks connecting one ligand resonance to another in the bound state can be selectively detected, providing bound ligand conformations (transfer nuclear Overhauser effect experiments). More recently, paramagnetic perturbations are being used to locate ligand-binding sites and determine glycan conformations. Although there are some notable cases of structure deposition using the above methods (61), and even some exploiting related methods of solid-state NMR (62), there are many more structures that are better described in words, pictures, and molecular dynamics movies. The latter cases are no less important and rely no less on deposited structures of the underlying proteins.

**Figure 6:**
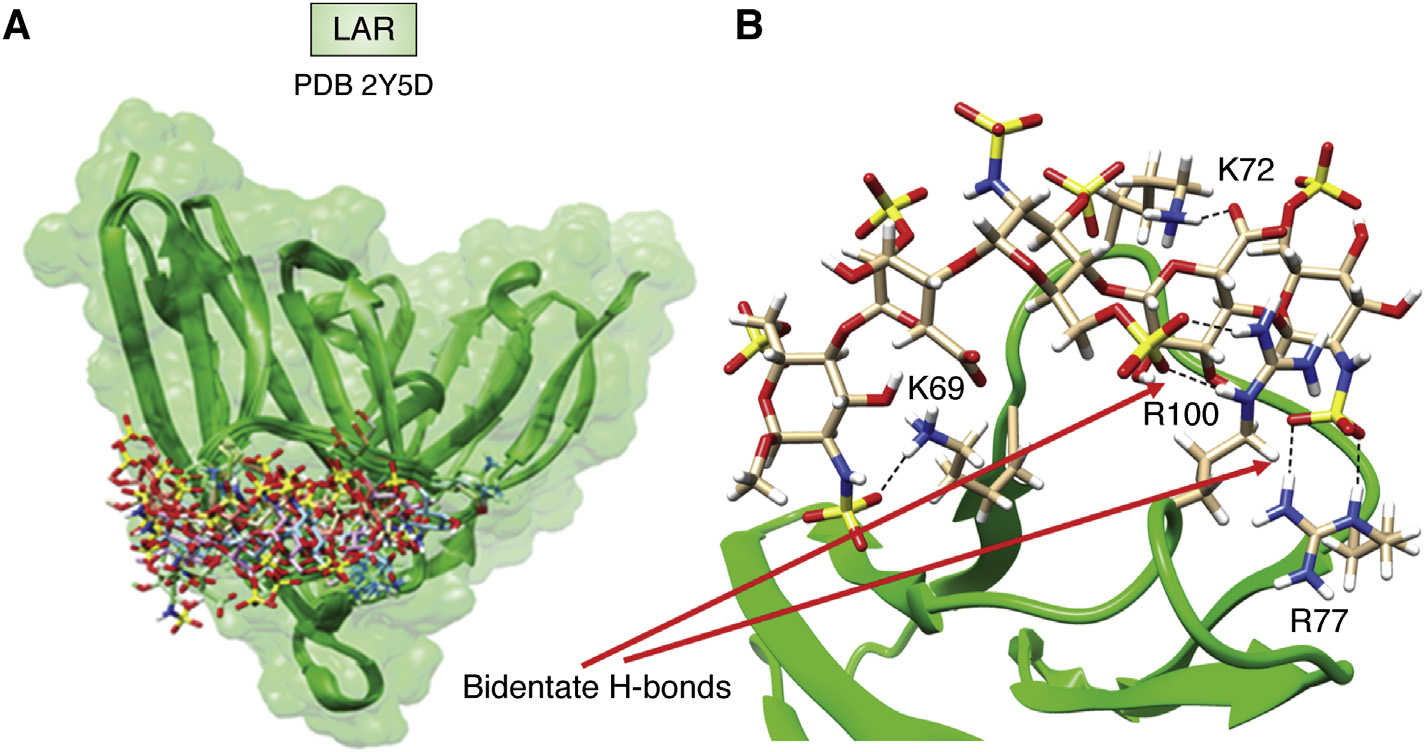
**Docked structures of LAR using PDB deposition 2Y5D.***A*, the cluster of docked HS structures. *B*, the snapshot from molecular dynamics refinement showing the bidentate hydrogen bond between R77 and N-sulfate oxygens of the terminal GlcNS residue, as well as that between R100 and O6 sulfate oxygens of the central GlcNS6S residue (*dotted lines*, indicated with *arrows*). LAR, leukocyte common antigen-related protein; PDB, Protein Data Bank.

One example drawn from my laboratory's own work involves a short piece of heparan sulfate (HS), actually the commercial anticoagulant, fondaparinux (GlcNS6S-GlcA-GlcNS3,6S-IdoA2S-GlcNS6S-OMe), bound to the terminal two domains of the leukocyte common antigen-related protein (LAR) (63). This system is of considerable interest because of its involvement in regulation of axon outgrowth and nerve regeneration after injury (64). Among the factors involved, is LAR’s competitive interaction with chondroitin sulfate and HS components of the glycocalyx that surrounds most human cells (65). There are more than a dozen crystal structures containing the two *N*-terminal domains of LAR, including one with sucrose octasulfate as an HS mimic (2YD8). While sucrose octasulfate and HS both have extensively sulfated sugar residues, the degree to which sucrose octasulfate mimics a native HS ligand is clearly in question. To produce a better model, we undertook an NMR study that included chemical shift perturbation, saturation transfer difference, and transfer nuclear Overhauser effect data. These data were used in a docking study that began with the unliganded crystal structure, 2YD5. The docking software, HADDOCK (66), was used to produce the cluster of structures shown in Figure 6*A*. HADDOCK can use a variety of experimental data as constraints, including those mentioned above, and it has become one of several platforms for integrating various types of data in the structural determination of large complexes (67).

Subsequent to publication, a low-energy structure that satisfied all experimental data was used as a starting point for a long (1 μs) molecular dynamics simulation. This allowed examination of the many structures sampled near this low-energy structure. A snapshot showing a frequent interaction between the negatively charged *N*-sulfate on the GlcNS residue at the nonreducing terminus of fondaparinux and the positively charged side chain of Arg 77 is shown in Figure 6*B*. This has a bidentate hydrogen bond involving 2 sulfate oxygens and 2 N-H groups of the arginine. A similar interaction occurs transiently between Arg 100 and the O-6 sulfate of the interior GlcNS. Additional stabilization comes from lysine side-chain interactions with other negatively charged groups of the ligand. Of course, there are many documented cases of lysine- and arginine-rich pockets binding negatively charged glycosaminoglycans such as chondroitin sulfate and HS. However, the structural details are intriguing, suggesting a search for structurally similar interactions that might occur in other systems.

Another example of how deposited structures, combined with computational docking of ligands, can provide important biological insight comes from recent work on the SARS-CoV-2 virus. The angiotensin-converting enzyme-2 is currently regarded as the cell-surface receptor for the virus. In fact, there is a crystal structure of the receptor-binding domain of the viral spike in complex with angiotensin-converting enzyme-2 (6M0J). However, Clausen *et al.* (68) noticed a cluster of positively charged residues near the interface of these two proteins that suggested a possible HS binding site and proceeded with docking studies to verify the interaction. These were followed by an extensive set of cell-based studies to convincingly document cell-surface HS as a required coreceptor for the virus. Thus, docking may suggest new molecular targets for disruption of virus–host cell interactions.

**Figure 7:**
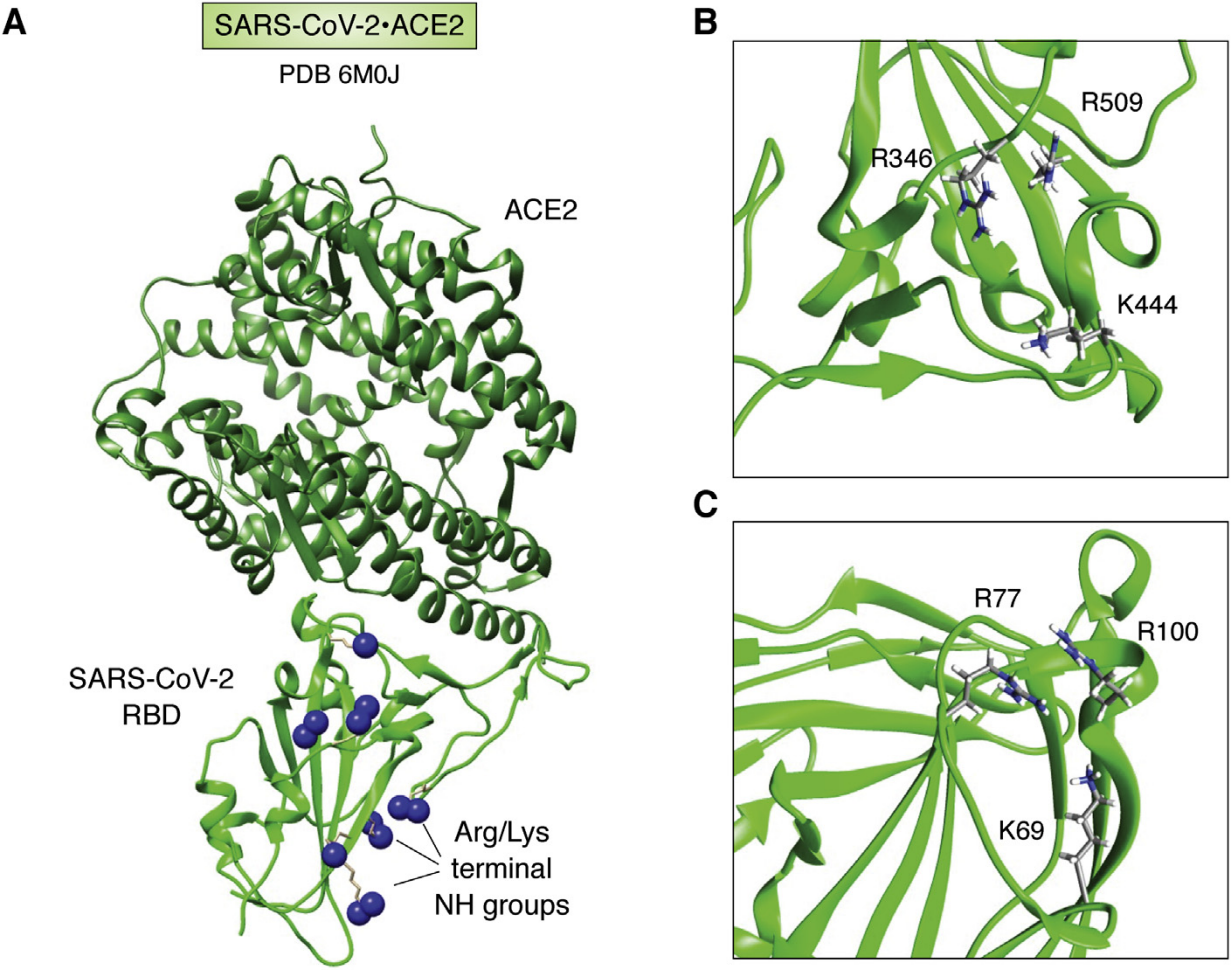
**HS-binding clusters in SARS-CoV-2 RBD and LAR.***A*, the complex of SARS-CoV2 RBD (*green*) with ACE2 (*forest green*) (6M0J). *Blue spheres* are terminal NH groups of arginines and lysines. *B*, expansion of SARS-Cov2-RBD showing charged clusters: R509, R346, and K444. *C*, expansion of LAR (2YD5) showing charged clusters R100, R77, and K69. HS, heparan sulfate; LAR, leukocyte common antigen-related protein; RBD, receptor-binding domain.

The region identified on the receptor-binding domain and the cluster of positively charged residues are depicted in Figure 7*A*. Figure 7, *B* and *C* present an interesting comparison of a subset of these residues to those implicated in the study of LAR binding to HS. Despite the very different sequence distribution of residues (K69, R77, R100 for LAR and K444, R346, and R509 for SARS-CoV-2), the triad of a lysine residue and two arginine residues have inter-residue separations of β-carbons that differ by less than 1.5 Å. Observations like this suggest that mining the PDB for structural details of binding sites could impact our ability to anticipate ligand-mediated cell–cell or cell–pathogen interactions in other systems. The future will tell if such suggestions have merit.

## Conclusion

Glycobiology has clearly benefitted from decades of structural deposition in the PDB. However, obstacles associated with preparation of samples having homogeneous glycans, as well as recognizing glycans when they are present in a deposited structure, may have diminished impact both within and outside the glycobiology community. Many of these obstacles have now been overcome. Annotation of deposited structures is much improved, there are new glycan-specific tools for searching the PDB, and symbolic representation of glycans is facilitating depiction of protein–glycan interactions in complex systems. New structural technologies, such as cryo-EM and solid-state NMR, which do not require crystallization, promise to provide many new glycan-containing structures. However, the primary goal of most glycobiologists, and structural biologists in general, remains a functional understanding. Structures of glycoproteins and glycan-binding proteins can provide a basis for this understanding, but integration of data relating to dynamics, energetics, and transient interactions will be required. Continued efforts to facilitate access to deposited structures by the broad range of scientists who integrate these data will be key to achieving this goal.

## Conflict of interest

The author declares that he has no conflicts of interest with the contents of this article.
